# Augmentation of the insufficient tissue bed for surgical repair of hypospadias using acellular matrix grafts: A proof of concept study

**DOI:** 10.1177/2041731421998840

**Published:** 2021-04-20

**Authors:** Debora Morgante, Anna Radford, Syed K Abbas, Eileen Ingham, Ramnath Subramaniam, Jennifer Southgate

**Affiliations:** 1 Jack Birch Unit for Molecular Carcinogenesis, Department of Biology and York Biomedical Research Institute, University of York, Heslington, York, UK; 2 Hull York Medical School, Heslington, York, UK; 3 Paediatric Urology, Leeds Teaching Hospitals NHS Trust, Leeds General Infirmary, Leeds, UK; 4 Central Biomedical Services, University of Leeds, Leeds, UK; 5 School of Biomedical Sciences, Institute of Medical and Biological Engineering, University of Leeds, Leeds, UK

**Keywords:** Acellular matrix, biomaterial, surgery, hypospadias repair, tissue integration

## Abstract

Acellular matrices produced by tissue decellularisation are reported to have tissue integrative properties. We examined the potential for incorporating acellular matrix grafts during procedures where there is an inadequate natural tissue bed to support an enduring surgical repair. Hypospadias is a common congenital defect requiring surgery, but associated with long-term complications due to deficiencies in the quality and quantity of the host tissue bed at the repair site. Biomaterials were implanted as single on-lay grafts in a peri-urethral position in male pigs. Two acellular tissue matrices were compared: full-thickness porcine acellular bladder matrix (PABM) and commercially-sourced cross-linked acellular matrix from porcine dermis (Permacol™). Anatomical and immunohistological outcomes were assessed 3 months post-surgery. There were no complications and surgical sites underwent full cosmetic repair. PABM grafts were fully incorporated, whilst Permacol™ grafts remained palpable. Immunohistochemical analysis indicated a non-inflammatory, remodelling-type response to both biomaterials. PABM implants showed extensive stromal cell infiltration and neovascularisation, with a significantly higher density of cells (*p* < 0.001) than Permacol™, which showed poor cellularisation and partial encapsulation. This study supports the anti-inflammatory and tissue-integrative nature of non-crosslinked acellular matrices and provides proof-of-principle for incorporating acellular matrices during surgical procedures, such as in primary complex hypospadias repair.

## Introduction

Decellularised tissue matrices offer a promising natural biomaterial in surgical situations where there is either an inherent lack of a tissue bed for repair, or where the healthy tissue bed is compromised by trauma or fibrotic scarring. One such need is encountered in surgical repair for hypospadias. With reported frequencies of 0.3 to 7.0 per 1000 live births, hypospadias is one of the most common genitourinary birth defects, requiring revision in as many as 1 in 300 boys (reviewed^1,2^). Hypospadias is associated with the development of a foreshortened urethra resulting in an aberrantly-positioned external orifice (meatus) on the ventral aspect of the penis. Surgical repair is the mainstay treatment for the majority of infants with hypospadias, but can require multiple procedures and is frequently associated with unsatisfactory results and complications, including the formation of urethral fistulas, stenosis and dehiscence or rupture of the repair. The underlying pathophysiology of many complications is the inherent lack of healthy host vascularised tissue, with tension on these inadequate tissues resulting in poor healing and dehiscence of the wound.^3^ Retrospective reviews of patient outcome following two-stage repair for severe hypospadias have reported complication rates from 58 to 68%.^2,4,5^ Fistulas and strictures are particularly difficult to manage due to a lack, or poor quality of tissue at the site of repair.

Post-operative complications increase with the severity of the anomaly^6^ and patients with severe hypospadias often require extra tissue to repair the urethra (reviewed^1^). Autologous free tissue grafts have been used clinically for urethral reconstruction, including skin from genital and extra-genital regions,^7,8^ with buccal mucosa the most commonly used.^9–11^ Problems most commonly associated with the use of autologous free tissue grafts include graft size, donor site morbidity and graft contracture.^12^ Preclinical studies aimed at improving hypospadias repair outcomes by applying novel biomaterials and tissue-engineering techniques have met limited success (reviewed^2^). Such approaches have included the use of biomaterials of synthetic or natural derivation, either unseeded or cell-seeded in both flat and tubularised configurations in rabbits, dogs or rats (80 preclinical studies reviewed in Versteegden et al.^13^). A range of acellular biological scaffolds derived from allogeneic and xenogeneic sources using different decellularisation processes have been used in these pre-clinical studies including porcine small intestinal submucosa (SIS, Surgisis^®^), Alloderm^®^, decellularised bladder submucosa and decellularised urethra.^13^ Due to a lack of controlled preclinical studies, however, the efficacy of these approaches has been difficult to determine. Although pre-clinical studies in animals have tended to suggest better results and reduced complications when acellular matrices are combined with cells, such findings have not been confirmed in the limited number of clinical studies (reviewed^13^). However, a major confounder is the tendency to test novel approaches clinically only after standard surgical procedures have failed.

The ideal biomaterial to enhance urethral tissue repair would provide a suitable template to replicate the biological and biomechanical functional properties of the host tissue by allowing progressive ingrowth of periurethral tissue components, without inducing an adverse host response that would lead to fibrosis and contracture. The size of the defect and the extent to which endogenous cells can infiltrate and organise within a graft material have been reported as key limitations. In male rabbits, a 0.5 cm tubularised decellularised matrix of porcine bladder submucosa was reported to be the maximum length able to support normal tissue formation.^14^ However, it is important to highlight that different decellularisation methods are utilised by different research groups and different protocols can have varying effects on the extent of cell and DNA removal, composition of the extracellular matrix and biomechnical attributes of the resultant biological scaffold matrix resulting in variations in the potential for constructive tissue remodelling.^15,16^

We have developed proprietry decellularisation processes using low concentration sodium dodecyl sulphate (0.1% (w/v) SDS) and proteinase inhibitors for the production of a range of porcine and human tissue specific biological scaffolds including cardiac valves,^17,18^ dermis,^19^ arteries^20^ and musculoskeletal tissues.^21,22^ Importantly, these processes preserve the biomechanical and biological tissue properties. Preclinical^23^ and clinical^24–27^ studies have clearly demonstrated the utility of this approach. We have adapted this process for full thickness porcine bladder to create an acellular porcine bladder biomaterial (PABM) particularly aimed at urological applications.^28^ Using an ex vivo model in which human urinary tract tissue was combined with PABM in organ culture, we have previously associated host M2-polarised CD163+ tissue macrophages with the pioneering events of cellular infiltration and integration at the tissue:decellularised biomaterial interface.^29^

It is reported that Permacol™ (aka Pelvicol™), a commercial cross-linked collagen acellular matrix derived from porcine dermis and licenced for surgical use, may reduce the complications of primary complex hypospadias repair when used as a peri-urethral graft.^3^ In the off-label study, it was suggested that the graft supported the urethroplasty as a splint, but there was no scope for the histological outcome to be assessed. Our previous in vitro studies have indicated that unlike PABM,^29^ Permacol™ lacks cell integrative properties.^30^ In order to help inform future clinical development, the aim of this study was to evaluate gross and histological outcomes of incorporating PABM or Permacol™ as peri-urethral grafts in an experimental large animal model. The male juvenile pig was chosen as the animal species because of the anatomical size and physiological similarity to male children. The cellular response to the implanted scaffolds at 3 months was studied using CD163 (M2 macrophages) with progenitor markers of haematopoietic (CD34), leucocyte (CD45) and myofibroblast (SMA) lineages to assess the cell-integrative properties of non-cross-linked and cross-linked natural biomaterial matrices.

## Materials & methods

### Biomaterials

For PABM production, pig bladders were collected from a local abattoir (Traves & Son Ltd, Escrick) on ice in Transport Medium consisting of Hank’s balanced salt solution (Gibco) containing 10 mm HEPES pH 7.6 (Gibco) and 20 kallikrein inhibiting units/ml aprotonin (Trasylol^®^, Nordic Pharma).^31^ PABM, a full-thickness porcine acellular bladder matrix, was produced aseptically using the decellularisation procedure described by Bolland et al.,^28^ including a terminal disinfection stage with peracetic acid. PABM was batch-tested by histology and Hoechst 33258 staining of sections from multiple samples to confirm the absence of cells or double-stranded DNA. Contact cytotoxicity tests conducted in vitro as described^28^ confirmed both sterility and absence of toxic by-products.

Permacol™ was purchased from Medtronic (Watford, UK).

### Animal husbandry, analgesia and anaesthesia

Large White Landrace Hybrid male pigs 14 weeks old of approximately 15–20 kg weight were ear-tagged for identification and housed in pairs with unlimited access to water. Assessment of the animals was performed at least twice daily and animals were weighed every two weeks.

All experimental procedures were approved by the local Animal Welfare and Ethical Review Body and were conducted at the University of Leeds animal surgical facility under a project licence granted by the UK Home Office, in accordance with the Animal Scientific Procedures Act 1986. Details to fulfil the essential and recommended ARRIVE 2.0 guidelines for reporting animal studies is available as Supplementary Information.

Food was withheld 16 h prior to surgery. Initial sedation was performed using intramuscular Midazolam 0.32 mg/kg (Hypnovel Roche, UK) and Azeperone 2.25 mg/kg (Stresnil Elanco Animla Health). An over-the-needle cannula (18 g Venflon) was inserted and secured in an ear vein. In some animals, where combination of Midazolam and Azeperoine did not produce enough sedation to allow intravenous catheterisation, 2.5% isoflurane in oxygen was delivered via a snout mask for no more than 1 min attached to an anaesthetic machine. This deepened the state of sedation enough to allow intravenous catheterisation of an ear vein. Following intravenous catheterisation, general anaesthesia was induced by intravenous injection of Propofol 4.0 mg/kg or to effect (Propofol Plus, Zoetis UK Limited). A 7–8 mm ID endo-tracheal tube (Sims Portex Limited) was introduced and anaesthesia maintained using Isofluorane (2.0–3.0% in oxygen).

An eye lubricant was applied and the skin was prepared for aseptic surgery using 5% Chlorhexidine (Vetasept, Animalcare Limited). A long acting antibiotic injection, Amoxicillin 15 mg/kg body weight (Ampoxypen LA 150 mg/ml Suspension for Injection, MSD Animal Ltd.) and a non-steroidal anti-inflammatory drug, Carprofen 4 mg/kg body weight (Rimadyl small animal solution for injection, Zoeist UK Ltd.) were given subcutaneously as separate injections before the start of the surgery. During the surgical procedure, 0.9% NaCl (Vitevax 1 9 mg/ml, Dechra Veterinary Products) was infused via the ear vein cannula at a rate of 40 ml/kg body weight.

At the end of the surgical procedure, 3 ml of local anaesthetic (0.5% Marcaine, AstraZeneca UK) was infiltrated locally and an opioid analgesic Buprenorphine 20 μg/kg body weight (Vetergesic 0.3 mg/ml solution for injection, CEVA Animal Health Ltd) was administered as intramuscular injection to provide postoperative pain relief. Further post-operative analgesia was dependent on animal behaviour and was provided either by Buprenorphine or Carprofen alone.

Humane euthanasia involved sedation with Midazolam and Azeperone as described above followed by an overdose of barbiturate (phentobarbital sodium 200 mg/ml solution; Euthatal, Merial Animal Health Ltd).

### Implantation of PABM and Permacol™ as on-lay urethral free graft

Surgery was performed on 12 pigs (mean weight 16.59 kg ±1.25 SD), with six animals receiving implants of PABM and six of Permacol™. The study was designed so that procedures were carried out on half the animals (three PABM and three Permacol™) followed by a 5 month gap in order to enable the first series to be analysed and inform the second series.

Under anaesthesia and complete aseptic conditions, a 5 cm midline incision was made caudally, approximately 5 cm from the preputial sac. The peri-urethral plane was opened and a 3.0 by 1.5 cm^2^ graft of PABM or Permacol™ was positioned and secured with eight to ten dissolvable Vicryl™ (polyglactin 910; Ethicon) sutures, consistent with the surgical procedure reported in children.^32^ Two non-dissolvable polypropylene (Prolene™, Ethicon) sutures were placed at either end of the graft in the opened superficial fascia in order to mark the implant site. The rest of the superficial fascia was closed using Vicryl™ interrupted sutures. Skin closure was achieved using absorbable suture in a continuous closure. Two further Prolene™ sutures were placed as external markers of the closure to enable location of the implant site after 3 months.

Euthanasia was performed 3 months post-operatively as planned and, following external inspection, implants with surrounding tissues were removed for analysis. Guided by the marker sutures, incisions were made and the graft and surrounding tissues removed en bloc, extending from the subcutaneous fat to the deep aspect of the penile shaft. During tissue collection from the first cohort of pigs, free movement of the penile shaft within the sheath of surrounding fascia and peri-urethral tissues made it difficult to register the orientation of the graft in relation to the penile structures for histology. To overcome this during tissue collection from the second set of pigs, the penile shaft and surrounding tissues were clamped before cutting through these structures beyond the site of the clamps. Clamps were replaced by sutures once the tissue was removed from the animal and prior to fixation.

### Histology & immunohistochemistry evaluation: Qualitative and quantitative analysis

Harvested tissues were divided in two to enable separate fixation in 10% (v/v) formalin in phosphate buffered saline and in zinc salts. Equivalent samples of non-implanted PABM and Permacol™ were processed in parallel. Following fixation, tissues were processed routinely into paraffin wax and 5 µm sections were collected onto slides. Standard haematoxylin and eosin staining was performed to evaluate the position and histological appearance of grafts and peri-urethral tissues. Staining with DNA intercalating Hoechst 33258 (0.1 µg/ml) was performed to assess tissue and graft cellularity.

Immunohistochemistry was performed, in some cases on serial sections, to identify the nature of the cell populations surrounding or infiltrating the implants. For this purpose, antibodies were selected against CD34 (haematopoietic progenitor marker), CD45 (leucocyte/macrophage lineage marker), CD163 (monocytes/macrophages of an M2 tissue-remodelling phenotype), MAC387 (recently infiltrated macrophages) and anti-smooth muscle actin (SMA expressed by myofibroblasts and smooth muscle cells of vascular structures). Antibodies were selected on the basis of immunoreactivity against paraffin wax-embedded porcine tissues as listed in Table 1. All antibodies were titrated for use, with appropriate positive and negative (irrelevant and no primary antibody) controls included in all series. All antibody labelling was performed on zinc-fixed tissues without antigen retrieval, except in the case of anti-CD34, where formalin-fixed paraffin wax-embedded sections were used.

**Table 1.** Primary porcine-reactive monoclonal antibodies used for immunohistochemistry.

Antigen	Distribution	Antibody clone	Supplier	Concentration
CD34	Haematopoietic, vascular and other lineages	EP373Y	Abcam	1:1000
CD45	Leucocyte lineage marker	K252-1E4	Serotec	1:150
CD163	Expressed by monocytes and macrophages of M2 tissue-remodelling phenotype	2A10/11	Serotec	1:200
SMA	Vascular structures & myofibroblasts	1A4	Sigma	1:4000
MAC387	Recently tissue infiltrating monocytes and macrophages	MAC387	AbD Serotec	1:150

All immunohistochemistry was performed on serial zinc-fixed tissue sections with exception of anti-CD34 which was applied to antigen-retrieved tissue sections from formalin-fixed paraffin wax-embedded tissues processed in parallel.

For zinc-fixed tissue sections, blocking of all free avidin/biotin sites (kit from Vector Laboratories) and secondary antibody binding sites (10% (v/v) rabbit serum; Dako) was performed before incubation overnight at 4°C with primary antibody. The secondary antibody, biotinylated rabbit anti-mouse immunoglobulin (Dako) was pre-incubated with 10% (v/v) swine serum (Dako) to eliminate cross-reactivity with porcine tissue. Bound antibody was detected using the Vectastain^®^ ABC kit (Vector Laboratories), with 3,3′-diaminobenzidine (DAB; SigmaFAST™ 3,3′-diaminobenzidine tablets) as chromogen.

For formalin-fixed sections, endogenous peroxidase was blocked with 3% (v/v) hydrogen peroxide, then antigen retrieval was performed by microwave boiling in 1 mm ethylenediamine tetra-acetic acid (pH 8.0) for 10 min. Secondary antibody binding sites were blocked with 2.5% (v/v) horse serum (Dako) before incubation overnight at 4°C with primary (anti-CD34) antibody. Bound primary antibody was detected using an Amplifier™ antibody, followed by ImmPRESS™ Excel amplified horseradish peroxidase (HRP) polymer reagent and ImmPACT™ DAB EqV substrate as the chromogen (ImmPRESS™ Excel Amplified HRP Polymer Staining Kit; Vector Laboratories).

Following labelling, all sections were counterstained in Mayer’s haematoxylin, dehydrated and mounted in 1,3-diethyl-8-phenylxanthine (DPX; Sigma-Aldrich).

Immunohistology was analysed to characterise and quantify the extent and type of cellularisation and tissue integration versus host reaction to the two implanted biomaterials (PABM and Permacol™). The analysis focused on the extent of cellular integration (cell type and density), presence and extent of any encapsulation process, and the number and distribution of immunolabelled cells. For analysis, labelled slides were scanned on a Zeiss Axioscan Microscope and the resulting CZI image files were subjected to supervised semi-automated analysis using StrataQuest software (version 6.0.0.123) on the TissueGnostic image analysis platform (Vienna, Austria). The auto-detection function was used to set the colour intensity of the master marker (nuclear haematoxylin) and DAB label to identify the different cell type-associated markers. Five non-overlapping 0.1 × 0.1 mm^2^ regions of interest (ROIs) were defined within each implanted biomaterial (PABM and Permacol™) and nuclei were detected automatically within the five equal-sized ROIs. Following optimisation, the same conditions were applied to all image files. Raw data were imported into GraphPad Prism for statistical evaluation.

## Results

### Survival and health of surgical recipients

**Figure 1.**
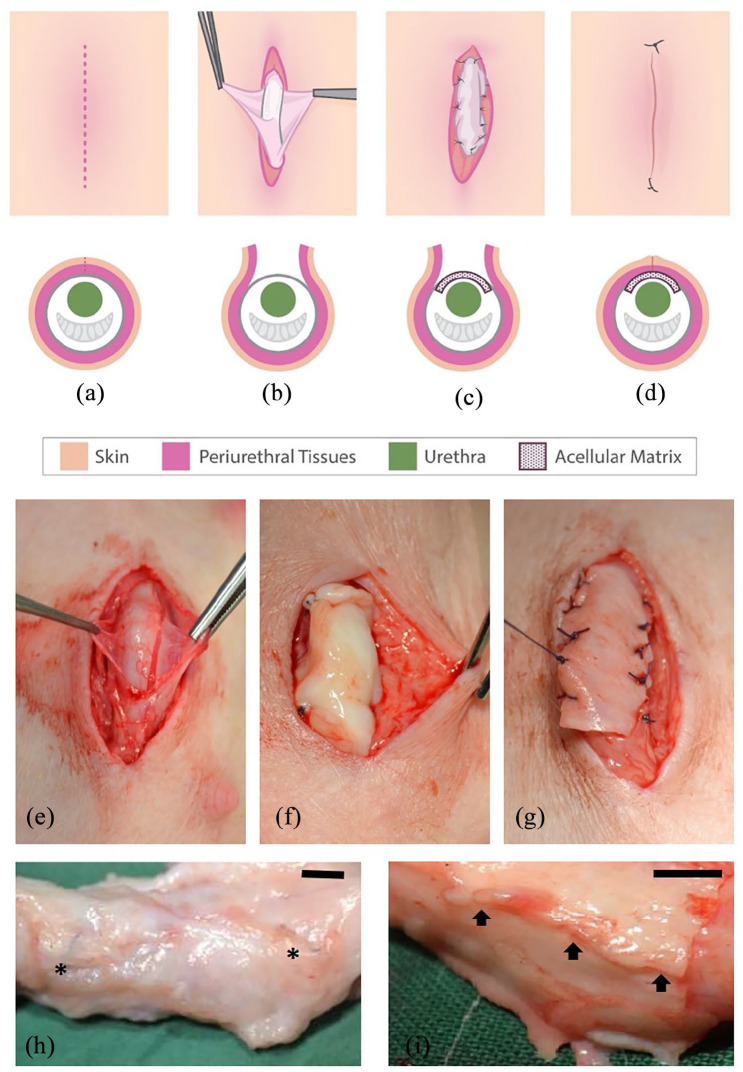
Surgical placement of biomaterial implant (PABM or Permacol™) within the peri-urethral fascia. Surgery was performed on 12 large white landrace hybrid male pigs 14 weeks old and mean weight of 16.59 kg (±1.25 (SD)). Six animals received implants of PABM and six of Permacol™: (a)–(d) Schematic of the surgical procedure. An incision (red dashed line in (a)) was made approximately 5 cm caudally from the preputial sac. The superficial adipose tissue was dissected (b) to reveal the peri-urethral tissues and fascia. The fascia was opened (b) in order that either PABM or Permacol™ could be sutured in place using eight Vicryl™ sutures, with two non-absorbable 3’0 Prolene^®^ marker sutures positioned at either end of the graft (c). Subcutaneous fat was then opposed followed by skin closure (d) achieved with 5’0 monocryl (ethicon) or Vicryl™ continuous suture with two external Prolene^®^ marker sutures at the caudal and cranial end of incision (d). (e)–(g) Intraoperative stages from surgery reflecting schematic parts (b) and (c) with insertion in part (c) of PABM (f) or Permacol™ (g). (h) and (i): At harvest, the grafted tissue area was removed ‘en-bloc’ for histological analysis using the delineating permanent marker sutures as guides for PABM (h) and Pelvicol (i). In (h) arrows highlight the persistence of Permacol™; in (i) asterisks highlight the position of permanent sutures used to mark the position of the graft. Scale bar 1 cm.

The procedure is illustrated schematically in Figure 1(a) to (d). Surgery proceeded according to plan with biomaterials positioned as onlay urethral grafts (Figure 1(e)–(g)). All pigs survived the immediate and long-term post-operative period with no complications; voiding was normal and there were no episodes of urinary retention, urinary tract or wound infections. Upon termination at 3 months, the body weight of the animals ranged from 55 to 62 kg (mean 56.12 kg). Gross anatomy, as examined at the time of dissection and harvesting of tissue around the graft, was similar to control animals, with no substantial scarring, fibrosis or encapsulation. Macroscopically PABM grafts appeared fully integrated and could only be identifed from the positioning of the non-absorbable marker sutures (Figure 1(h)), whilst Permacol™ grafts remained readily apparent (Figure 1(i)).

### Extent and patterns of cellularisation

**Figure 2.**
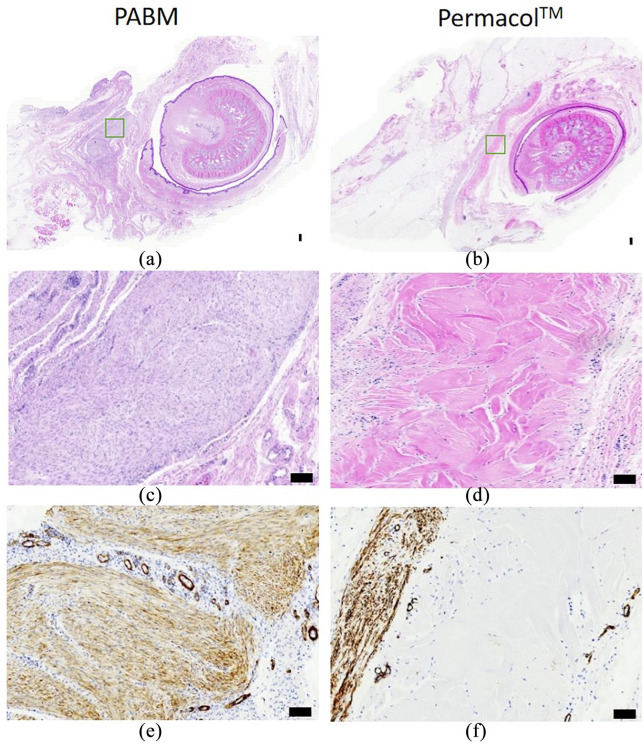
Histology of on-lay graft implants after 3 months, showing PABM (left column) or Permacol™ (right column): (a)–(d) H&E-stained sections of peri-urethral penile tissue showing PABM and Permacol™ implants at low power (a & b), with boxes (green) marking the position of implant regions illustrated at higher magnification in (c & d) (by H&E) and (e & f) (by IHC) to examine differences in the extent of cellularisation of each biomaterial. Note the extensive infiltration by cells in PABM (c) marked by haematoxylin-stained nuclei (blue dots), compared to the absence of cells across the Permacol™ graft (pink) in (d). Immunolabelling with anti-αSMA indicates differences in distribution of vessels and αSMA+ cells between PABM (e) and Permacol™ (f) implants. PABM implants showed extensive cellular infiltration and neovascularisation, whereas Permacol™ implants showed cells and vessels retained along the edges of the implant. Note partial encapsulation evident along one edge of Permacol™ (f).

Following dissection and histological evaluation, the biomaterial implants were identified in all 12 animals, as illustrated in Figure 2(a) and (b). Samples of non-grafted PABM or Permacol™ processed in parallel for haematoxylin and eosin (H&E) stain and immunohistochemistry revealed multidirectional collagen bundles and an absence of cells that provided a morphological reference for identifying the implanted grafts.

Histologically, there was no widespread inflammation associated with any graft and there was no detection of any MAC387^+^ cells, which would have been indicative of recently infiltrated macrophages. In one Permacol™ graft, a small localised reaction of giant cells within a thin capsule was found coincident with the Prolene™ marker suture and provided an internal control for the potential for foreign body reaction. In addition, occasional foci of lymphocytes were observed at the edges of Permacol™ samples which, based on location, frequency and distribution, related to the position of the Vicryl™ absorbable sutures used to attach the grafts.

Varying extents of cellularisation were apparent within the implant sections analysed from the 12 grafts. Implanted PABM grafts revealed cells present uniformly throughout the implant (Figure 2(c)). There was evidence of vascularisation at the periphery and within the graft. This was highlighted by αSMA immunolabelling, which was detected on vascular elements as well as by a majority of cells within the PABM grafts (Figure 2(e)). In the case of PABM implants, there were no lymphocytic aggregates found and no evidence of any encapsulation-like reaction around the periphery of the implant (Figure 2(f)). An absence of cellularisation was typical of Permacol™ grafts, where cells accumulated at the edge of the implant and showed very limited, sparse infiltration (Figure 2(d)). Where present, cells showed a tendency to infiltrate along natural pathways within the Permacol™ grafts, sparsely infiltrating between collagen bundles (H&E shown in Figure 2(d)). Evidence of a partial encapsulation-like reaction, where cells accumulated at the interface between the Permacol™ graft and host tissue, was apparent in some regions. This partial encapsulation reaction exclusive to the Permacol™ implants was particularly evident when sections were immunolabelled with antibodies to αSMA (Figure 2(f)). As measured from scans of all six Permacol implants, the capsule involved between 6 and 44% (min-max range) of the perimeter of the visualised implanted biomaterial (mean ± SD: 19.5% ± 12.59, *n* = 6). The average thickness of the identified capsule was 216 µm ± 96 (mean ± SD, *n* = 6; min–max range: 20–500 µm).

### The nature and abundance of infiltrating cell populations

**Figure 3.**
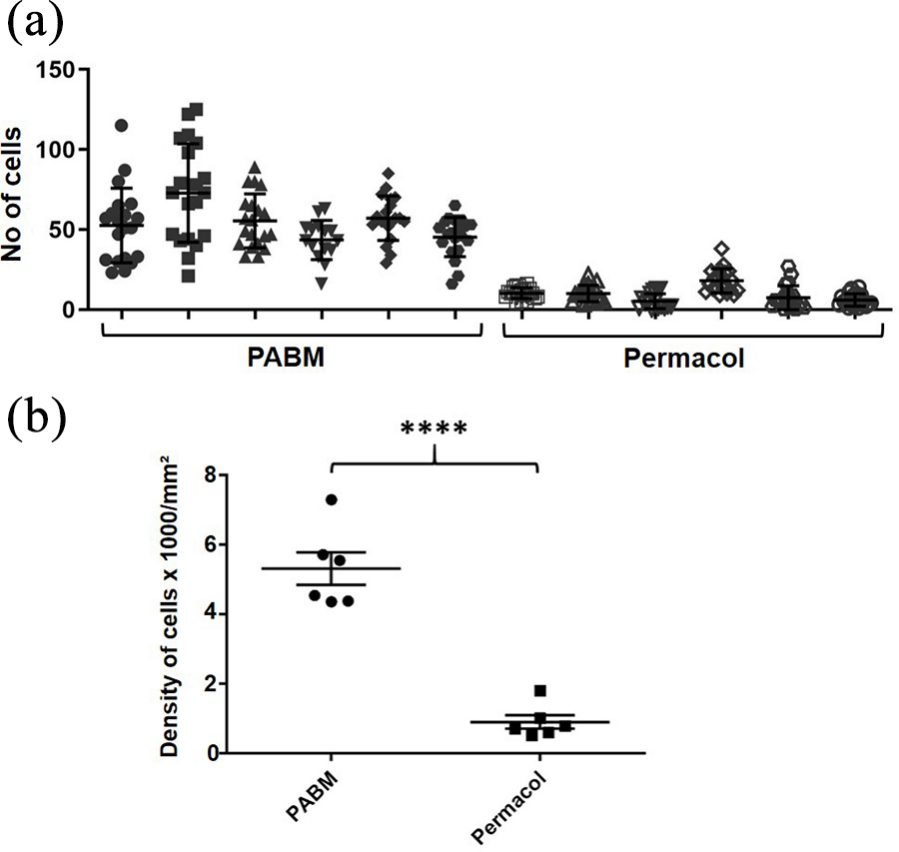
Implant cell density: (a) scatter plot for number of cells detected within the biomaterial implant for each animal displayed individually, showing mean and SD. Using tissue analysis software, nuclei were detected automatically within 20 non-overlapping equal-sized regions of interest (ROIs) for each PABM and Permacol™ implant. Data are displayed for the individual animals to show the variance within and across grafts. Filled symbols represent PABM data and empty symbols represent Permacol™ data and (b) combined data from all animals displayed as mean number of cells per mm^2^ (±SEM) in PABM versus Permacol™ (5309 ± 78 vs 906 ± 32; *p* < 0.0001; *n* = 6 animals per group).

An objective quantitative analysis of immunohistochemically-labelled tissue sections was carried out. Total cell counts confirmed significantly higher cell densities in PABM than in Permacol™ implanted grafts (Figure 3). The cell count per ROI is shown for each animal to illustrate the extent of variation between animals and across the grafts (Figure 3(a)). The density of infiltrating cells expressed as the mean total number of cells per mm^2^ ± SEM was 5309 ± 78 for PABM versus 906 ± 32 for Permacol™ (*n* = 6 animals per group; Figure 3(b)). This difference was statistically significant (*p* < 0.0001, determined using Welch’s t-test).

**Figure 4.**
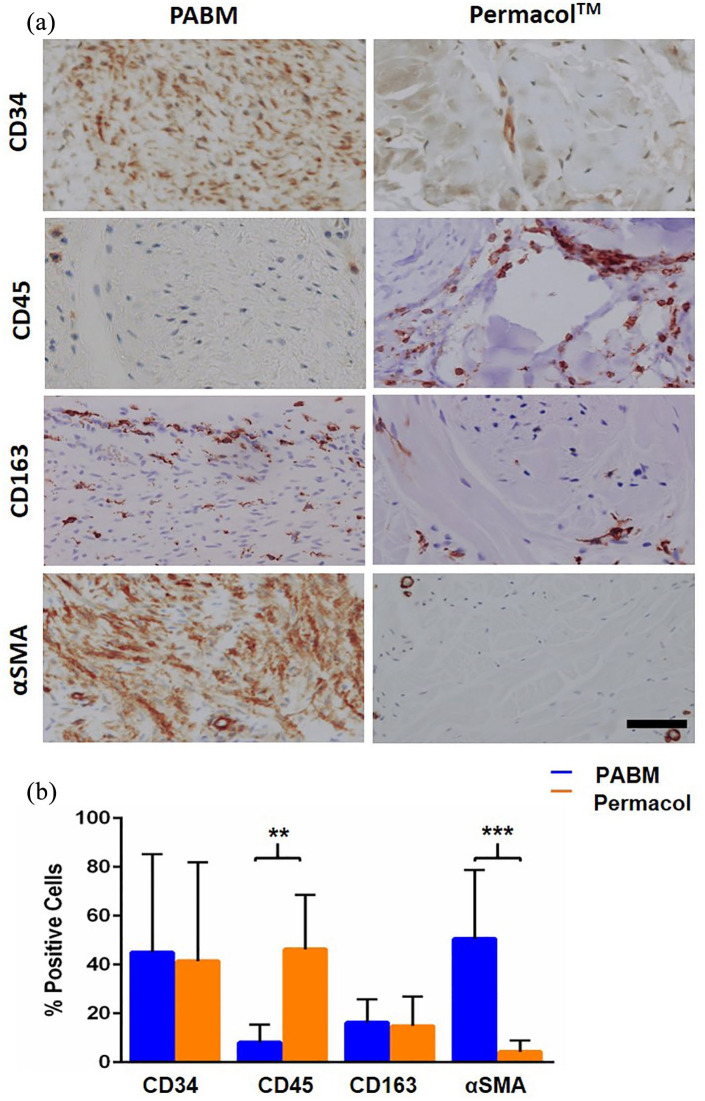
Distribution of infiltrating cell types in implants: (a) immunohistochemistry of Permacol™ and PABM implants labelled with antibodies against CD34, CD45, CD163 and αSMA to identify lineages of infiltrating host cell populations. Scale bar 100 µm and (b) quantification of the relative propotion of different cell types in PABM and Permacol™ samples identified using cell type lineage markers. Supervised automated image analysis using StrataQuest software was used to quantify cell numbers from 5 individual ROIs per implant for each marker. Data expressed as mean ± SD.

To determine if, separate from the total cell count, there were shifts in the relative proportions of the different major cell types infiltrating PABM and Permacol™, the percentages of cells expressing CD34, CD45, CD163 and αSMA was examined. Whereas percentages of cells expressing αSMA, CD45 and CD163 were assessed in serial sections, the antibody to CD34 required a different tissue fixation to be immunoreactive and hence was counted in equivalent but non-related areas. The relative quantification revealed 40% CD34: 20% CD163: 40% αSMA positive cells in PABM, compared to 40% CD34: 20% CD163: 40% CD45 positive cells in Permacol™. In other words, although both biomaterials were infiltrated by cells expressing CD34+ and/or CD163+ cells in a similar ratio, the remaining 40% of the infiltrating population showed a significant switch from predominantly CD45+ in Permacol™ to αSMA+ in PABM (Figure 4).

## Discussion

The aim of hypospadias repair is to provide a good cosmetic and functional outcome, resulting in a penis devoid of ventral curvature and the patient being able to pass urine from the tip of the penis with a good stream. Our study investigated the concept that an acellular matrix graft inserted into a peri-urethral position would become tissue-integrated, augmenting a deficient tissue bed on the ventral aspect and reducing the potential for surgical complications. By being positioned peri-urethrally, our approach differs in surgical approach from other studies where acellular matrices, either alone or seeded with cells, have been used as inlay grafts in urethral reconstruction in both animal and human studies (reviewed^13^).

In this present study aimed at acquiring proof-of-concept evidence towards clinical translation, we have demonstrated that implanting decellularised tissue matrices into the peri-urethral stroma in a large animal surgical model is well-tolerated and does not provoke an inflammatory response. We compared two porcine matrices that varied in their different surgical handling characteristics from highly compliant (PABM) to stiff (Permacol™). The handling differences are supported by mechanical evidence, as the Young’s modulus for Permacol™, reported to be 50–100 MPa,^33^ is considerably higher than the 2–3 MPa we found for native porcine bladder,^34^ albeit that some stiffening occurs following decellularisation.^28^ Nevertheless, how different tissue derivations and/or processing affects biomaterial properties and influences implantation outcomes is an incomplete science. Bladder and dermal matrices both contain collagens type I and III, but whereas type III collagen is associated in the bladder with healthy compliance,^35^ in the dermis it is associated with rigidity and scarring,^36^ reflecting negative implications for (dermal) scaffold design.^37^ This highlights the need for further basic studies to inform intelligent scaffold design, alongside empirical, translation-focused studies of the type reported here.

Externally, both porcine-derived biomaterials used gave acceptable results. Nevertheless, there were important biological differences in the host response to the two materials. Implants of PABM had become fully incorporated within the three-month period to leave no macroscopic residue. Histologically, the marked PABM graft region was extensively vascularised and completely infiltrated by cells. This agrees with independent reports of non-cross-linked matrices in terms of superior host tissue integration and cellular infiltration accompanied by neovascularisation^38,39^ By contrast, Permacol™ implants persisted macroscopically and the bulk material remained acellular at 3 months. Permacol™ is terminally-sterilised by gamma radiation and chemically cross-linked by hexamethylene diisocyanate,^40^ the latter producing stable urea groups by the interaction of amine groups with isocyanate.^41^ Cross-linking is known to limit scaffold degradation^5^ and to inhibit host cells from infiltrating matrix grafts, including Permacol™. Although we might predict acellular matrix properties to reflect tissue-specific differences, it seems axiomatic that observed differences in results between Permacol™ and PABM were dominated by the influence of cross-linking. Supporting this point, we have shown previously that cells fail to infiltrate PABM cross-linked by gamma-radiation.^29^

Cells expressing αSMA+ were present both as vascular smooth muscle cells in association with blood vessels and as spindle-shaped myofibroblasts. The myofibroblasts were present either within the PABM or in the case of Permacol™, at the neo-vascularised interface of the biomaterial and native host tissue where they formed an incomplete capsule-like structure. An encapsulation response to Permacol™ has been described in some studies,^38,42^ albeit with exceptions.^43^ The myofibroblast is recognised as an essential effector of both healthy tissue regeneration through matrix remodelling and pathological fibrosis, although how this balance is regulated is not fully understood (reviewed^44,45^). Our observations suggest that the physical cross-linking of the matrix might define whether recruited myofibroblasts formed a boundary at the edge of the implant or infiltrated the matrix.

Given the difference in extent of cellular infiltration into Permacol™ and PABM, we were interested in whether there was any relative shift in infiltrating cell types. Historically, any evaluation of cellularisation in implanted biomaterials has been performed using qualitative or semi-quantitative approaches. In the field of histology and immunohistochemistry, it is well known that different observers see and report the same tissue sample differently.^46–48^ To overcome this limitation, we performed an objective quantitative analysis of immunohistochemically-labelled biomaterials 3 months post-implantation. Although restricted by the limited availability of reliable porcine-reactive antibodies, we were nevertheless able to examine the major infiltrating cell lineages as identified by expression of CD34 (to identify progenitor cells of multiple lineages, particularly haematopoietic (reviewed^49^)); CD45 (as a pan-leucocyte marker); CD163 (macrophages of M2 phenotype) and αSMA (vascular and other smooth muscle cells, including myofibroblasts). These different markers revealed that after 3 months, both implanted biomaterials contained a similar proportion of CD34+ and CD163+ cells, making up about 60% of the total cellular infiltrate. The presence of CD163-expressing cells further indicated that both acellular collagen matrices promoted a remodelling, regenerative (M2), rather than inflammatory (M1) macrophage response, as observed previously with PABM in human ex vivo studies.^29^ The remainder 40% infiltrating population was different between matrices, being constituted by spindle-shaped αSMA+ cells in PABM versus diffuse lymphoid CD45+ cells in Permacol™. This reveals key differences in recellularisation biology and outcome between the two matrices, discussed below.

Irrespective of matrix tissue derivation (bladder or dermal) or cross-linked status, neither acellular matrix tested provoked an acute reactive or rejection response upon implantation. Nevertheless, the presence of a diffuse infiltrating CD45+ population in Permacol™ was indicative of a low level, chronic inflammatory state, as was reinforced by the presence of enhanced acute local reactions at permanent and resorbable suture sites. Thus, although implanted acellular matrices were not themselves inflammatory and promoted an M2-type macrophage phenotype, the PABM was the more benign material, possibly as a result of being non-cross-linked. The absence of innate immune-activating signals in PABM fits with our previous observations using a novel ex vivo human tissue:PABM interface model, where early (<10 days) ‘pioneering’ cellular events involved recruitment of tissue-resident macrophages and polarisation to an M2 CD163+ remodelling phenotype.^29^ The ability of PABM as a non-cross-linked allogeneic material to promote a fully integrative response perforce rests on the quality of matrix production, including absence of innate immune-inducing ‘danger’ signals such as DNA. This clearly needs stringent quality-control during batch production if the material is to be taken forward for clinical use. This also raises the question of sterilisation, as most terminal sterilisation methods involve cross-linking, such as by gamma-radiation, which our previous results indicate may impact negatively on tissue integrative properties.^29^

The availability of PABM and Permacol™ as biomaterials with contrasting properties of host integration/remodelling versus persistence may suit different surgical applications. The process of chemical cross-linking results in a product that remains rigid but flexible and resistant to degradative processes and therefore maintains strength and 3D structure. This resilience of Permacol™ was exploited by Springer and Subramaniam to support urethral repair where Permacol™ was incorporated as a peri-urethral splint in 12 boys undergoing urethrocutaneous fistula repair (10) or redo urethroplasty (2).^32^ Apart from one instance of late wound infection, no recurrence of fistula or stricture was noticed in the cohort at a median follow up of 2.5 years. The results supported the principle of managing complications from hypospadias surgery by incorporating a suitable biomaterial into the surgical procedure when local tissues are insufficient or inadequate. The clinical nature of the study meant that outcomes were observational only, with no possibility of obtaining follow-up histological evidence of the extent or nature of any tissue integration. Our present study highlights the importance of studying histological outcomes in a clinically-relevant in vivo model, as the eventual fate of the cross-linked implant material in the clinical study^32^ is unknown. On a related point, our study demonstrates that even in the case of natural non-cross-linked tissue matrices, the complete remodelling and integration events take place over a longer timescale than the 3 months typically reported for in vivo models.

In addition to its superior integration, other benefits of PABM are its compliance, malleability and ability to hold sutures. In accordance with the recognised ideal characteristics of a biomaterial for urethral use (reviewed^50^), PABM provided a functional strong and supple scaffold when placed in the peri-urethral plane in a simple porcine urethroplasty model. From its characteristics, it is predicted that in primary hypospadias repair, where insufficient native tissue is available, PABM may fulfil an unmet surgical and clinical need as a scaffold for augmentation of the native tissue in order to prevent subsequent complications, such as urethral fistulae and strictures.

In conclusion, this study provides proof of principle evidence that implanted non-crosslinked acellular matrices become readily incorporated, meaning they may be useful to augment an inadequate tissue bed to support primary surgical repair. In the particular case of hypospadias, the successful incorporation of an onlay graft of acellular matrix during the primary surgery may improve the quality of repair, leading to reduced complications. It is clear that natural acellular tissue matrices offer promising new biomaterials to support reconstructive and regenerative surgery, but that many of their advanced physical and biological properties are negatively affected by cross-linking as a result of conventional chemical- or radiation-induced terminal sterilisation procedures. If natural acellular matrices are to meet their full clinical potential it is important to evaluate the safety/efficacy outcomes from both ex vivo^29^ and in vivo studies and use these to define new criteria for the regulated production of such materials for clinical use.

## Supplemental Material

sj-docx-1-tej-10.1177_2041731421998840 – Supplemental material for Augmentation of the insufficient tissue bed for surgical repair of hypospadias using acellular matrix grafts: A proof of concept study Click here for additional data file. Supplemental material, sj-docx-1-tej-10.1177_2041731421998840 for Augmentation of the insufficient tissue bed for surgical repair of hypospadias using acellular matrix grafts: A proof of concept study by Debora Morgante, Anna Radford, Syed K Abbas, Eileen Ingham, Ramnath Subramaniam and Jennifer Southgate in Journal of Tissue Engineering
