# Supplementary material for: Augmentation of the insufficient tissue bed for surgical repair of hypospadias using acellular matrix grafts: A proof of concept study
Source: J Tissue Eng. 2021 Apr 20;12:2041731421998840. doi: 10.1177/2041731421998840 (PMC8060745; doi:10.1177/2041731421998840)
Supplement: sj-docx-1-tej-10.1177_2041731421998840 – Supplemental material for Augmentation of the insufficient tissue bed for surgical repair of hypospadias using acellular matrix grafts: A proof of concept study [file sj-docx-1-tej-10.1177_2041731421998840.docx]

ARRIVE guidelines 2.0. https://arriveguidelines.org/

|  | **Essential 10** |  |
| --- | --- | --- |
| 1a | The groups being compared | We developed a porcine experimental model of urethroplasty to study the effect of incorporating an onlay free graft of acellular matrix at the repair site. We compared two acellular matrices: 1) PABM (Porcine Acellular Bladder Matrix a non-crosslinked, full thickness matrix) and 2) Permacol™ (a commercial porcine dermis-derived acellular matrix that is licensed for human use and has previously been used as an off-label product in a small clinical series undergoing hypospadias repair). |
| 1b | The experimental unit | Single animal |
| 2a | Experimental units allocated to each group and total number in each experiment; total number of animals used | Twelve large white hybrid (LWH) pigs were used in the experimental study with a total of six animals implanted with PABM and six with Permacol. |
| 2b | How sample size was decided | A maximum of 6 pigs could be housed at any one time |
| 3a | Inclusion or exclusion criteria and data points during the analysis | Only male pigs were used - both sexes of animal were not used because the study was targeted for a condition that does not occur in females.  All animals included in the study remained healthy and none were excluded at any stage of the study |
| 3b | Any animals not included in the analysis and why. | NA |
| 3c | Report exact value of n in each group | 12 animals total in 2 groups of n=6 each. |
| 4a | Randomisation of animals to control and treatment groups | Six animals were implanted with PABM and six with Permacol™. The animals were divided into two groups of six with three pigs having PABM and the other Permacol™ implanted into the peri-urethral tissues. The surgery for first and second groups took place ~five months apart.  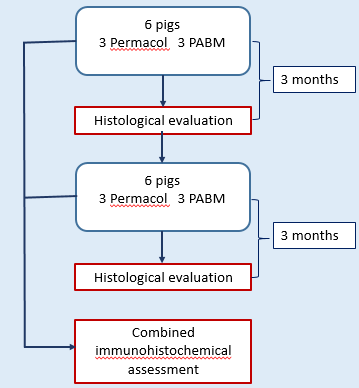 |
| 4b | Strategy to minimise experimental confounders | Surgery was performed in two batches of 6 to limit  any inherent bias that may have ensued by implanting only Permacol™ or PABM first, thereby improving the surgical procedure used for the second batch of six. |
| 5a | Blinding – who was aware of group allocation during allocation, conduct, outcome and data analysis | To perform blinded analysis, labelled slides were scanned on a Zeiss Axioscan Microscope and the resulting CZI image files were subjected to semi-automated supervised analysis using StrataQuest software (version 6.0.0.123) on the TissueGnostic image analysis platform (Vienna, Austria). Five non-overlapping 0.1 x 0.1 mm^2^ regions of interest (ROIs) were defined within each implanted biomaterial (PABM and Permacol™) and nuclei were detected automatically within the five equal-sized ROIs. Following optimisation, the same conditions were applied to all image files. |
| 6a | Define outcome measures | Weight gain; Normal voiding function; External palpability; Histological evaluation of capsule formation; inflammation; cellularisation by density & cell type. |
| 7a | Statistical methods and software | Raw data were imported into GraphPad Prism for statistical evaluation using descriptive statistics. |
| 8a | Species, strain, gender, age, weight | Large white hybrid (LWH) male animals averaging 16.59 kg ± 1.25. |
| 9a | Experimental procedures – what, where, when and why | During the surgical implantation of the grafts, the peri-urethral plane was opened. The graft was secured with eight dissolvable Vicryl™ (polyglactin 910; Ethicon)  sutures. Two Prolene® (polypropylene; Ethicon) non-dissolvable “marker” sutures were placed at either end of the opened superficial fascia to the graft to enable localisation of the implant site. The rest of the superficial fascia and subcutaneous fat (where necessary) was closed using Vicryl™ interrupted sutures. Skin closure was achieved using Vicryl™ or Monocryl® in a continuous closure. Two further Prolene® sutures were placed as external markers of the closure to enable successful location of the site at 3 months. |
| 10a | Summary/descriptive stats for each experimental group plus variance | Upon termination at three months, the body weight of the animals ranged from 55-62 kg (mean 56.12 kg).  In the Permacol group only, there was a partial encapsulation reaction involving between 6% and 44% (min-max range) of the perimeter of the visualised implanted biomaterial (mean ±SD: 19.5% ± 12.59, n=6). The average thickness of the identified capsule was 216 μm ± 96 (mean ± SD, n=6; min-max range: 20 - 500 μm).  The density of infiltrating cells expressed as the mean total number of cells per mm^2^ ± SEM was 5309 ± 78 for PABM versus 906 ± 32 for Permacol™ (n=6 animals per group; p < 0.0001 using Welch t test).  Relative quantification revealed 40% CD34: 20% CD163: 40% αSMA positive cells in PABM, compared to 40% CD34: 20% CD163: 40% CD45 positive cells in Permacol™. Thus, although both biomaterials were infiltrated by cells expressing CD34+ and/or CD163+ cells in a similar ratio, the remaining 40% of the infiltrating population showed a significant switch from predominantly CD45+ in Permacol™ to αSMA+ in PABM. |

|  | **Recommended set** |  |
| --- | --- | --- |
| 11 | Abstract | Acellular matrices produced by tissue decellularisation are reported to have tissue integrative properties. We examined the potential for incorporating acellular matrix grafts during procedures where there is an inadequate natural tissue bed to support an enduring surgical repair. Hypospadias is a common congenital defect requiring surgery, but associated with long-term complications due to the poor quality/quantity of underlying tissue bed at the repair site.  Biomaterials were implanted as single on-lay 3.0 by 1.5 cm^2^ grafts of PABM or Permacol™ in a peri-urethral position in male pigs. Two acellular tissue matrices were compared: full-thickness porcine acellular bladder matrix (PABM) and commercially-sourced cross-linked acellular matrix from porcine dermis (Permacol™). Anatomical and immunohistological outcomes were assessed 3 months post-surgery.  There were no complications and surgical sites underwent full cosmetic repair. PABM grafts were fully incorporated, whilst Permacol™ grafts remained manipulatable. Immuno-histochemical analysis indicated a non-inflammatory,  remodelling-type response to both biomaterials. PABM implants showed extensive stromal cell infiltration and neovascularisation, with a significantly higher density of  cells (p< 0.001) than Permacol™, which showed poor cellularisation and partial encapsulation.  This study supports the anti-inflammatory and tissue-integrative nature of non-cross-linked acellular matrices and provides proof-of-principle for incorporating acellular matrices during surgical procedures, such as in primary complex hypospadias repair. |
| 12a | Background, rationale and experimental approach | Background  There is a clinical need in urology to identify biomaterials that can be used for reconstructive surgery of the lower urinary tract, including the bladder and urethra. Hypospadias is one of the most common birth defects in males (1 in 300) and is associated with development of an abnormal urethra. Surgical repair is performed for a majority of infants with hypospadias, but repair of severe hypospadias may require multiple procedures and is frequently associated with unsatisfactory results, including the formation of urethral fistulas in up to 20% of cases. These fistulas may be persistent and difficult to manage due to a lack of, or poor quality, tissue at the site of repair. Success rates for fistula repair with multiple attempts is unsatisfactory (between 66.6% and 92%) and recurrence rates are highest with simple closure.  Rationale  Our rationale was that natural biomaterials may support surgical repair and if incorporated in primary hypospadias cases could reduce the incidence of complications.  Experimental procedures.  Anaesthesia  Food but not water was withheld for 18 hours prior to surgery. Initial sedation was performed using intramuscular Hypnovel (0.3 mg/kg) (Roche) and Stresnil (1.2 mg/kg)  (Elanco). The animal was left for 20-30 minutes in a quiet and calm environment before transportation to the anaesthetic room on a trolley. Induction of anaesthesia was achieved by utilising a snout mask containing an isoflurane-soaked. Isoflurane 2.5 % in oxygen was then used for maintenance of anaesthesia and delivered via the snout mask attached to an anaesthetic machine. The electrocardiogram, pulse, blood  pressure and oxygen saturation of the animal was continuously monitored during the surgical procedure. Eyes were protected using Lacri-lube® (Allergan-Actavis).  An over-the-needle intravenous cannula (22 G) was introduced into an ear vein and secured with Micropore ™ (3M). The animal was given AmoxyPen LA (MSD Animal  Health) (15 mg/kg) and Rimadyl (Zoetis) (2 mg/kg), a non-steroidal anti-inflammatory agent  Positioning for surgery  The anaesthetised animal was place supine on the operating table. Skin on the lower abdomen was shaved. A plate electrode (Conmed) was placed onto shaved flank skin and attached to the diathermy machine. The animal’s skin was prepared with Chlorhexidine solution (Vetasept-Animal Care). The animal was draped using 307 x 254cm StreriDrapes with  incise pouch (3M). The monopolar diathermy pen (Ambu) was attached to the diathermy machine.  Surgical Basics:  A 5cm midline incision was made, approximately 5cm from the preputial sac, caudally using a size 15 disposable scalpel (Swann Morton). The peri-urethral tissues were opened using blunt dissection. Bleeding was stopped, when necessary using  monopolar diathermy.  Post-operative care:  At skin closure 3ml of 0.5 % Marcaine local anaesthetic was infiltrated locally. In addition, Vetergesic (0.3 mg), an opioid analgesic was administered to provide postoperative  pain relief. The animal was transferred from the operating table to trolley and then to a pen with fresh bedding and sawdust. The animal was allowed to recover in a quiet environment in isolation until up on all four limbs and drinking water. Once this was achieved the animal was placed in a pen with another animal and food made available.  Schedule 1  The process of euthanasia began with sedation of the animal using Hypnovel and Stresnil. An ear vein was cannulated and barbiturate was administered intravenously to overdose. |
| 12b | Relevance to humans | In order to be relevant to children, a large surgical model was required to test surgical compatibility and provide an adequate model in terms of anatomical size. |
| 13 | Objectives | The aim was to develop a large experimental model equivalent to anatomical size to children, in which to test surgical compatibility and examine the potential use of PABM in paediatric urology. The following experimental objectives were investigated:  - To determine the cellular integration properties of PABM when surgically implanted in vivo as a free onlay graft in a peri-urethral position  - To compare the integration of non-crosslinked PABM with that of a commercially-available crosslinked decellularised dermal matrix (Permacol™) |
| 14 | Ethical statement | All experimental procedures were approved by the local Animal Welfare and Ethical Review Body and were conducted at the University of Leeds animal surgical facility under a project licence granted by the UK Home Office, in accordance with the Animal Scientific Procedures Act 1986 under project licence (PPL70/7930). |
| 15 | Housing and husbandry | Large White Hybrid (LWH) male pigs were transported to the animal surgical facility for seven days quarantine. Numbered ear tags were used to aid individual identification. The animals were inspected for signs of disease by a vet and the facility’s technical staff and animals were housed together in pens, with appropriate clean bedding. |
| 16 | Animal care and monitoring | All animals were monitored and evaluated at least twice/day. The animals were weighed every two weeks over the full period and feeding regime altered accordingly. Food was available up to twice a day, with the amount varying according to weight gain and behaviour. Access to water was unlimited. |
| 17 | Interpretation/scientific implications | Implanting an acellular tissue matrix into the peri-urethral stroma in a large animal surgical model is safe and does not provoke an inflammatory response. Superficially, both the porcine-derived biomaterials used gave outwardly acceptable results. Nevertheless, there were important biological differences in the host response to the two materials. Implants of PABM had become fully incorporated within the three-month period to leave no macroscopic residue. Histologically, the marked PABM graft region was extensively  vascularised and completely infiltrated by cells. This agrees with independent reports of non-cross-linked matrices in terms of superior host tissue integration and cellular infiltration accompanied by neovascularisation. By contrast, Permacol™ implants persisted macroscopically and the bulk material remained acellular at three months. This is consistent with other studies using cross-linked biomaterials, including changes we ourselves have noted following PABM crosslinking. |
| 18 | Generalisability/translation | The experiments were performed in a surgical model of relevant scale and provide support to the principle of managing complications from hypospadias surgery by incorporating a suitable biomaterial into the surgical procedure when local tissues are insufficient or inadequate. By providing histological evidence of the extent and nature of tissue integration outcomes when different biomaterials are used, the results add important insight to small scale clinical studies where outcomes have been observational only. |
| 19 | protocol registration | NA |
| 20 | Data Access | Access to the raw histological qualitative and quantitative data required to reproduce the findings is available on request from the corresponding author. |
| 21 | Declaration of interests | The work was partially funded through the Medical Technologies Innovation and Knowledge Centre (phase 2 - Regenerative Devices), funded by the EPSRC under grant number EP/N00941X/1 as Proof of Concept awards: PoC023 and PoC045. AR was supported by the European Society of Paediatric Urology. JS is supported by a programme grant from York Against Cancer. The work leading to the development of PABM was originally funded by the Biotechnology and Biological Sciences Research Council (BBSRC) on grants E20352 and BB/E527220/1.  Eileen Ingham is a shareholder and consultant to Tissue Regenix Group PLC. The authors confirm that there are no other known conflicts of interest associated with this publication and there has been no significant financial support for this work that could have influenced its outcome. |
